# Survival probability and under-five mortality predictors in Western Kenya between 2015 and 2020

**DOI:** 10.1186/s12889-025-25052-6

**Published:** 2025-11-10

**Authors:** Harun Odhiambo Owuor, Asito Stephen Amolo, George Aol, Fredrick Onduru, Richard Omore, Beth Tippett Barr, Victor Akelo, Stephen Munga, Dickens Samwel Omondi Aduda

**Affiliations:** 1 Kenya Medical Research Institute Center for Global Health Research, PO BOX 1578-40100, Kisumu, Kenya; 2 Jaramogi Oginga Odinga University for Science and Technology, Bondo, Kenya; 3 Liverpool School of Tropical Medicine, Kisumu, Kenya; 4 Nyanja Health Research Institute, Salima, Malawi

**Keywords:** Under-five mortality, Kenya, Africa, Survival probability, Mortality predictors

## Abstract

**Background information:**

Despite gains in reducing under-five mortality in Kenya from 52 to 41.6 deaths per 1000 live births between 2015 and 2022, the rate remains notably higher than the Sustainable Development Goal (SDG) 3 target of 25 deaths per 1000 by 2030. The impact of interventions on risk factors and socio-structural inequities have varied across regions, indicating potential effects of unaddressed issues. There is a need to further understand the key drivers of under-5 mortality in given contexts to help improve intervention effectiveness.

**Objective:**

To estimate the survival probability and determine predictors of under-five mortality in Siaya County.

**Methods:**

The study utilized secondary data from the Siaya Health Demographic Surveillance System from 2015 to 2020; semi-annual population-based longitudinal surveillance, and data analyzed in cohort design. We performed both descriptive and multivariable analyses. We used the Cox regression model to estimate survival probability and predictors of under-five mortality. We employed an adjusted hazard ratio (AHR) to measure association. Significance was declared at *p* < 0.0.

**Results:**

There were 24,452 live births in the study period with 1,540 (6.3%) children dying before their fifth birthday. The cumulative survival probability among children under five was 92% with children of younger age being at increased risk of death. In Cox multivariable analysis, the risk of under-five mortality increased with decreasing maternal education (aHR 1.93; 95%CI1.19, 3.10) and number of Antenatal Clinic (ANC) visits (aHR 2.1; 95%CI 1.14, 1.62). Delivery at home/way to the hospital (aHR 1.51; 95% CI 1.29, 1.77); Maternal age less than 18 years (aHR 1.34; 95% CI 1.11, 1.62); no latrine (aHR 1.22; 95% CI 1.04, 1.43) and male gender were associated with reduced survival.

**Conclusion:**

Under-five mortality in Siaya was 63/1000 live births, which is higher than the national average of 41.6/1000 live births. Gender, maternal education, number of ANC visits, maternal age, delivery in hospital, and availability of toilets at home were the main predictors of under-five mortality. These observations reinforce the need to target multiple components at the community and institutional levels to reduce childhood mortality. Such interventions may target increasing uptake of preventive healthcare and education attainment.

**Supplementary Information:**

The online version contains supplementary material available at 10.1186/s12889-025-25052-6.

## Introduction

 Under-five mortality remains a prominent issue of public health concern with most of the early childhood mortality being due to conditions that could be prevented or treated with access to simple and affordable interventions [1]. Globally, approximately 4.9 million deaths were reported among under-five children in 2022. Sub-Saharan Africa accounted for 56% of these, an increase from 50.2% in 2015 [2]. In Kenya, under-five mortality has significantly declined from 101.5 deaths per 1000 live births in 1990 to 41.6 deaths per 1000 live births in 2020, driven by improved health services, immunization, bed net use, and free maternal and child healthcare [3]. The country still falls short of the SDG targets, with persistent regional and income disparities impacting maternal and child mortality indicators in the 47 countries [4].

Several studies have revealed mixed findings on various predictors of child mortality [5–7]. High maternal and/or community-level illiteracy, multiple births, low birth weight, short birth interval, low socio-economic status of households, and healthcare facility access challenges have been known to increase mortality. This demonstrates a complex interplay of diverse multi-level factors in under-five mortality across Africa [8–10]. Most of these studies are cross-sectional designs, such as national surveys and mega-data analyses collected after 5 to 10 years or more [11–13]. Data from longitudinal studies have not been frequently employed although they may provide a better association between the exposure and the outcome, enabling a better understanding of causality and dynamic of risk factors over time. It also minimizes recall bias and allows for evaluating life course influence on child survival [14].

Studies have shown the survival rate among under-five to be more than 70%, with high mortality experienced during the early neonatal period [15, 16]. A study conducted in selected countries in Sub-Saharan using Health Demographic Survey data in 2018 demonstrated that the cumulative survival rate was 88.9% among children below the age of five [17]. However, there is a paucity of data on the survival probability among those under the age of five years in Siaya County. The study aimed to estimate the survival probability and predictors of under-five mortality in Siaya County, Western Kenya.

## Methods

### Study design

Siaya HDSS is a system of continuous longitudinal monitoring of demographic events in a geographically defined population, with timely production of data on all births, deaths, and migrations. HDSS rounds gather information on births, deaths, pregnancies, migrations, morbidity, parent survival status, immunization, educational status, religion, marital status, and ethnicity semiannually. HDSS collects verbal autopsy data to determine causes of death among the HDSS population. They use the collected data to regularly track and measure demographic and health dynamics, including birth rates, mortality rates, causes of death, sickness, migration, and socioeconomic indicators. It also serves as a platform to monitor infectious and non-infectious disease indicators and compute disease rates by providing accurate denominators [18]. This study used a retrospective longitudinal cohort study design. In order to answer the objective of this study, we retrospectively extracted data (between 2015 and 2020) from the existing DSS database with our event of interest being under-five mortality recorded within the scope of five years.

### Study area

Siaya Health Demographic Surveillance System (HDSS) is located in Rarieda, Siaya, and Gem sub-counties in Siaya County, Western Kenya. The county is a malaria-endemic zone with a high HIV prevalence of 21% and an under-five mortality rate of 67.4 deaths per 1000 live births [19] which is more than the national average.

### Data source

The study utilized secondary data from the HDSS dataset downloaded from the KEMRI server and maintained in the Microsoft SQL database at the KEMRI Center for Global Health Research in Kisumu. The KEMRI/CDC HDSS follows a population of approximately 256,000 individuals, 42% of whom are children. These residents live in 393 villages spread over 700 km2, in 64,317 households and 44,461 compounds. The HDSS population was visited twice annually during the study period and data on basic demographic and health information were collected by community health interviewers.

### Study population

The study population for the analysis included all children under five years of age born within the study period and whose data was recorded in the Siaya HDSS database between 2015 and 2020. The child may be alive or dead at the time of the interview.

### Inclusion and exclusion criteria

We included all children between 0 and 59 months who were recorded in the HDSS database between 2015 and 2020. We excluded children whose data were incomplete from the analysis.

### Data management and analysis

The selection of covariates analyzed in this study was based on the associated literature review and the data missingness in the dataset. Exploratory data analysis was performed to evaluate the extent of missing values, identify potential outliers, and check for multicollinearity. Since children are the basic unit of analysis, data was transformed such that each child constituted a unit of observation. Follow-up time was censored to 59 months of age, death, or out-migration. Descriptive statistics were used to describe maternal and child characteristics. Cox bivariate proportional hazard model was fitted for outcomes against the predictors independently to identify the confounding and adjusted variables for the final model. A p-value of < 20% was used to identify the covariates to adjust for in the full model. Variables violating proportional hazard assumptions were excluded unless biologically plausible or supported by the literature. Covariates with > 10% missingness were excluded **(Supplementary 1)**, while those with < 10% were imputed using the multivariate imputation by chain equation (MICE). The final Cox proportional hazards model included variables with p-values < 0.05 and biologically plausible factors, reporting hazard ratios (HRs) at *p* < 0.05. We conducted the analysis using R version 4.2.1.

## Results

### Descriptive statistics of the study population

**Fig. 1:**
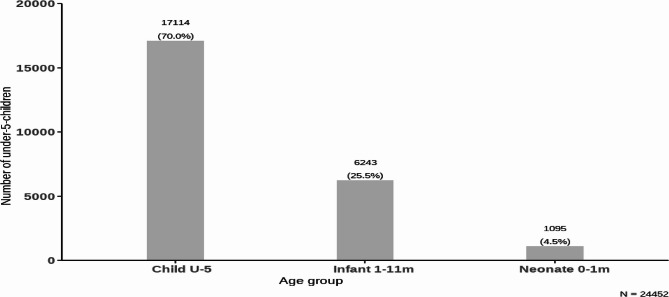
Distribution of under-five children by age group in HDSS, Siaya County, Kenya from 2015 to 2020

**Fig. 2:**
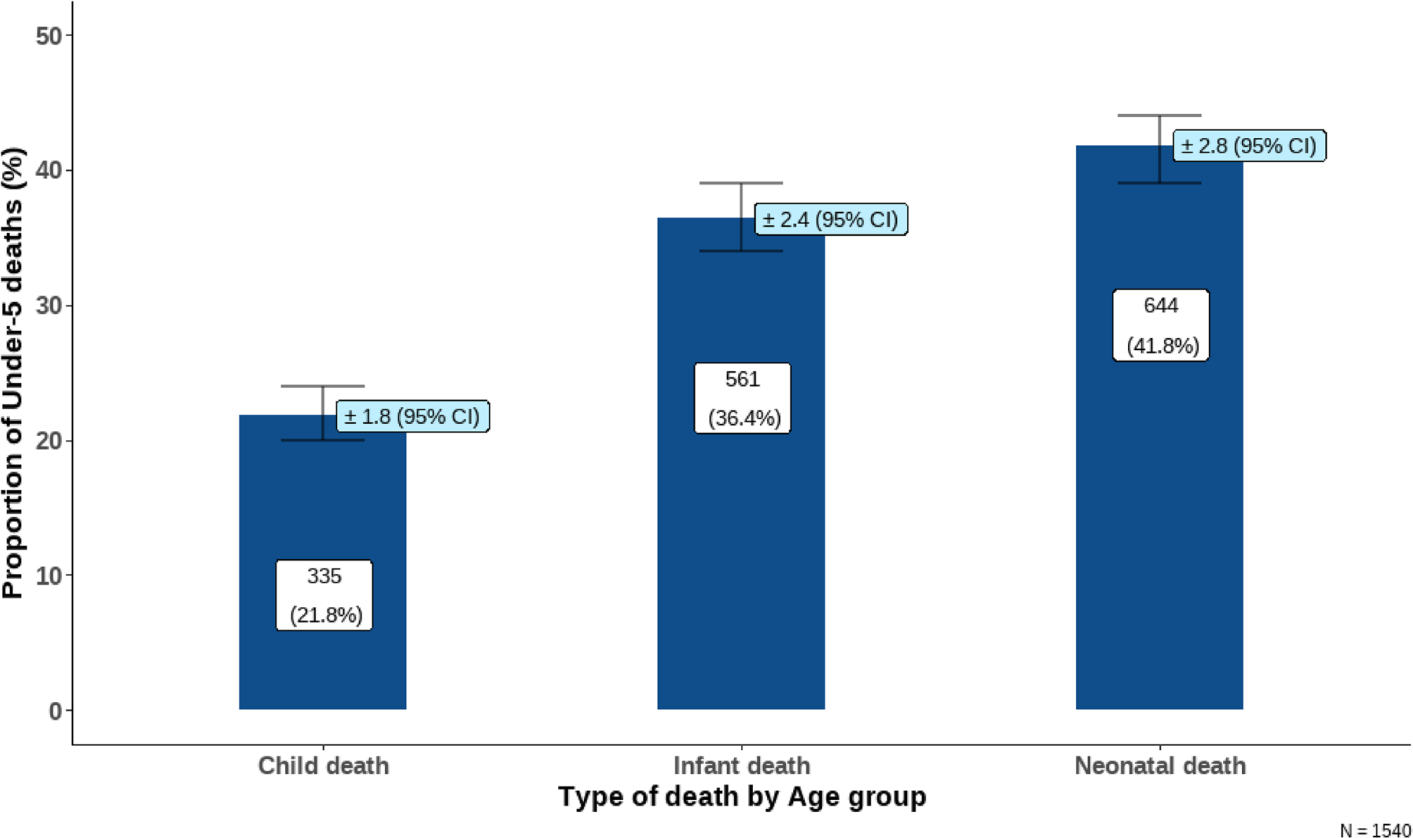
Deaths among children by age group in HDSS, Siaya County, Kenya from 2015 to 2020

A total of 24,452 children were observed during the study period, we excluded 7 children whose data was incomplete due to incomplete interviews. Most of the population was aged between 1 year to < 5 years **(**Fig. 1**)**. At the end of the follow-up, 1540 (6.3%) deaths were recorded, or 63 deaths per 1000 live births. The age distribution of participants is shown in Fig. 1 and deaths in Fig. 2, respectively. Different age clusters classified under-five child mortality as neonatal deaths (0–28 days), infant deaths (28 days to < 1 year), and child deaths - deaths between 1 year to < 5 years of age **(**Fig. 2**)**.

The male to female ratio was 1:1. Among children born to mothers under 18 years of age, 43.8% out-migrated, while 8.1% experienced under-five mortality. Under-five mortality was observed in 10.2% of children whose mothers received no antenatal care (ANC) and 8.1% of those whose mothers attended only one ANC visit. Additionally, 10% of children born at home or on the way to the hospital experienced under-five mortality. 12% of children born to mothers with no formal education experienced under-five mortality (Table 1).

**Table 1:** Under-five mortality by basic epidemiological and healthcare-related factors among participants in Siaya County, Kenya using HDSS data from 2015–2020

Characteristic	Overall, *n* (%)	Alive, *n*(%)	Outmigration, *n*(%)	Under-five Mortality, *n*(%)
Total	24,452 (100)	16,914 (69.2)	5,998 (24.5)	1540 (6.3)
Gender				
Male	12,341 (100)	8,556 (69.3)	2,933 (23.8)	852 (6.9)
Female	12,111 (100)	8,358 (69.0)	3,065 (25.3)	688 (5.7)
Maternal age				
Less than 18 years	1,809 (100)	869 (48.0)	793 (43.8)	147 (8.1)
18–34 years	19,075 (100)	12,984 (68.1)	4,951 (26.0)	1,140 (6.0)
Greater than 34 years	3,568 (100)	3,061 (85.8)	254 (7.1)	253 (7.1)
Family size				
1–3 members	7,365 (100)	4,539 (61.6)	2,272 (30.8)	554 (7.5)
4–6 members	11,755 (100)	10,421 (88.7)	750 (6.4)	584 (5.0)
7 + members	1,964 (100)	1,792 (91.2)	93 (4.7)	79 (4.0)
Wealth Index				
1 st quantile	3,710 (100)	2,360 (63.6)	1,092 (29.4)	258 (7.0)
2nd quantile	4,200 (100)	2,936 (69.9)	994 (23.7)	270 (6.4)
3rd quantile	4,164 (100)	2,903 (69.7)	1,016 (24.4)	245 (5.9)
4th quantile	3,889 (100)	2,747 (70.6)	901 (23.2)	241 (6.2)
5th quantile	3,753 (100)	2,537 (67.6)	1,006 (26.8)	210 (5.6)
Place of birth				
Home/way to hospital	2,131 (100)	1,355 (63.6)	543 (25.5)	233 (10.9)
Private hospital	4,087 (100)	3,095 (75.7)	821 (20.1)	171 (4.2)
Government hospital	18,189 (100)	12,438 (68.4)	4,619 (25.4)	1,132 (6.2)
Place of ANC Visit				
Gov. Dispensary	2,813 (100)	2,218 (78.8)	473 (16.8)	122 (4.3)
Gov. Health center	3,916 (100)	3,042 (77.7)	718 (18.3)	156 (4.0)
Gov. Hospital	15,539 (100)	10,171 (65.5)	4,229 (27.2)	1,139 (7.3)
Home	13 (100)	9 (69.2)	2 (15.4)	2 (15.4)
Private/mission	1,239 (100)	978 (78.9)	215 (17.4)	46 (3.7)
Others	8 (100)	7 (87.5)	1 (12.5)	0 (0.0)
ANC Times				
None	108 (100)	63 (58.3)	34 (31.5)	11 (10.2)
One	689 (100)	450 (65.3)	183 (26.6)	56 (8.1)
Two	1,893 (100)	1,263 (66.7)	466 (24.6)	164 (8.7)
Three	5,351 (100)	3,740 (69.9)	1,288 (24.1)	323 (6.0)
Above 4	14,731 (100)	10,468 (71.1)	3,407 (23.1)	856 (5.8)
Maternal Education level				
None	184 (100)	129 (70.1)	33 (17.9)	22 (12.0)
Primary	15,168 (100)	10,690 (70.5)	3,393 (22.4)	1,085 (7.2)
Secondary/high	7,629 (100)	5,077 (66.5)	2,178 (28.5)	374 (4.9)
Post-secondary	1,334 (100)	918 (68.8)	366 (27.4)	50 (3.7)
Religion				
Protestants	4,246 (100)	2,886 (68.0)	1,122 (26.4)	238 (5.6)
Catholic	5,584 (100)	3,815 (68.3)	1,441 (25.8)	328 (5.9)
Roho/legio maria	5,120 (100)	3,587 (70.1)	1,174 (22.9)	359 (7.0)
Others	9,181 (100)	6,379 (69.5)	2,210 (24.1)	592 (6.4)
Source of Water				
Protected water	7,465 (100)	5,077 (68.0)	1,937 (25.9)	451 (6.0)
Unprotected water	16,884 (100)	11,767 (69.7)	4,036 (23.9)	1,081 (6.4)
Water for Drinking				
Tap water	4,306 (100)	2,935 (68.2)	1,122 (26.1)	249 (5.8)
Lake/river	6,094 (100)	4,250 (69.7)	1,396 (22.9)	448 (7.4)
Rainfall,	3,986 (100)	2,833 (71.1)	952 (23.9)	201 (5.0)
Unprotected spring/borehole/well	6,117 (100)	4,209 (68.8)	1,510 (24.7)	398 (6.5)
Protected spring	3,159 (100)	2,142 (67.8)	815 (25.8)	202 (6.4)
Distance to Water Source				
In compound	4,985 (100)	3,514 (70.5)	1,231 (24.7)	240 (4.8)
< 500 m	13,976 (100)	9,721 (69.6)	3,342 (23.9)	913 (6.5)
500 m-2 km	4,783 (100)	3,189 (66.7)	1,251 (26.2)	343 (7.2)
2–5 km	186 (100)	131 (70.4)	42 (22.6)	13 (7.0)
> 5 km	10 (100)	5 (50.0)	5 (50.0)	0 (0.0)
Dk	291 (100)	204 (70.1)	71 (24.4)	16 (5.5)
Cooking methods				
Charcoal/firewood	23,915 (100)	16,559 (69.2)	5,837 (24.4)	1,519 (6.4)
Gas/paraffin/stove	537 (100)	355 (66.1)	161 (30.0)	21 (3.9)
Toilet type				
Neighbor/no facility/bush	2,375 (100)	1,590 (66.9)	584 (24.6)	201 (8.5)
Modern latrine	1,936 (100)	1,302 (67.3)	548 (28.3)	86 (4.4)
Traditional pit	19,921 (100)	13,873 (69.6)	4,810 (24.1)	1,238 (6.2)
Birth assistance				
Nurse/midwife/doctor/chw	22,870 (100)	15,926 (69.6)	5,584 (24.4)	1,360 (5.9)
Traditional birth attendant	1,548 (100)	970 (62.7)	401 (25.9)	177 (11.4)

*HDSS* Health Demographic Surveillence System ^*1*^ n, Mean (SD) ^*2*^ n (%)

### Survival probability of children under five years in Siaya County

**Fig. 3:**
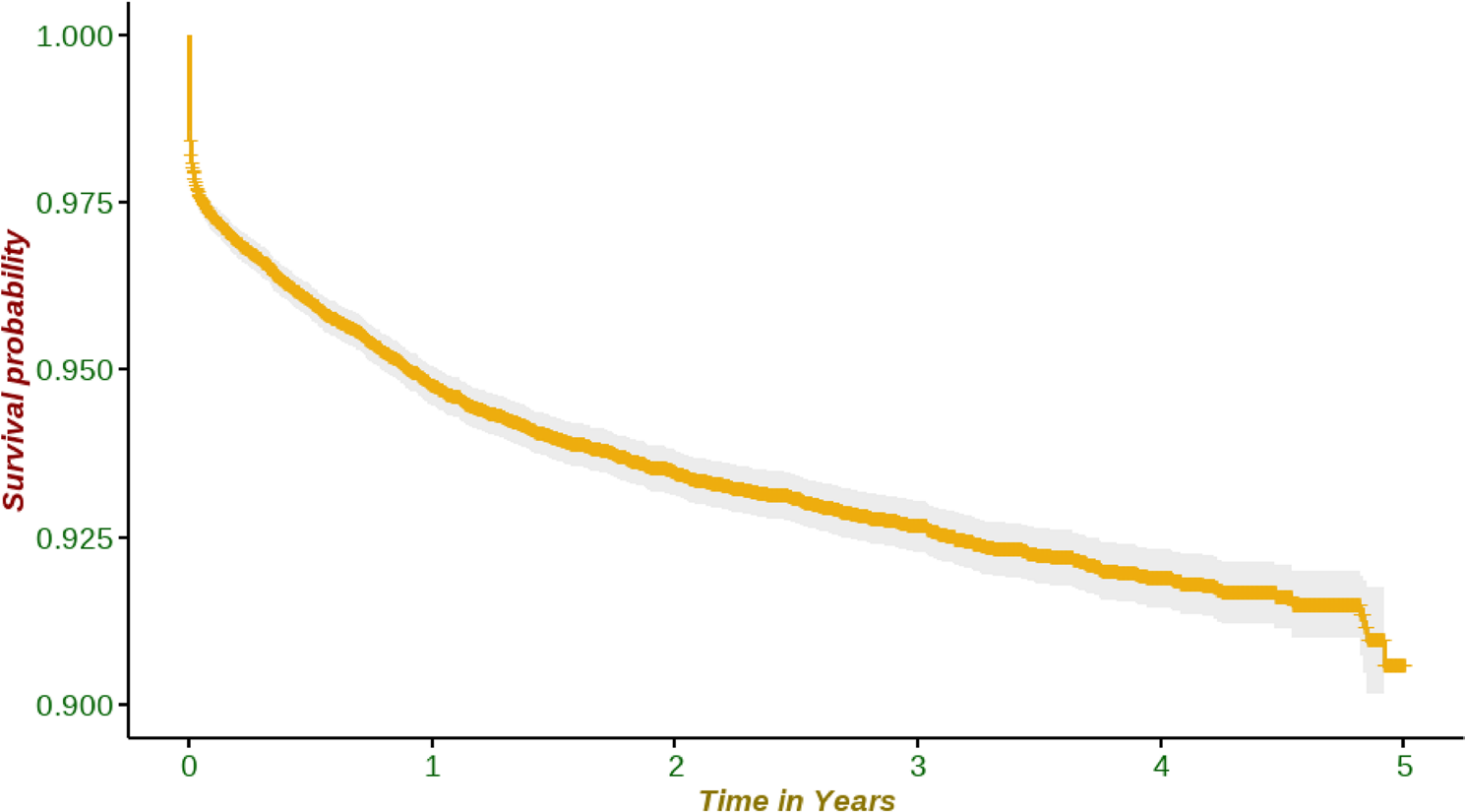
Survival probability of children under five in Siaya County using 2015–2020 HDSS data

The cumulative survival probability within the first five years was approximately 90% among the children born in the study area **(**Fig. 3**)**.

### Kaplan-Meier curve estimate

**Fig. 4:**
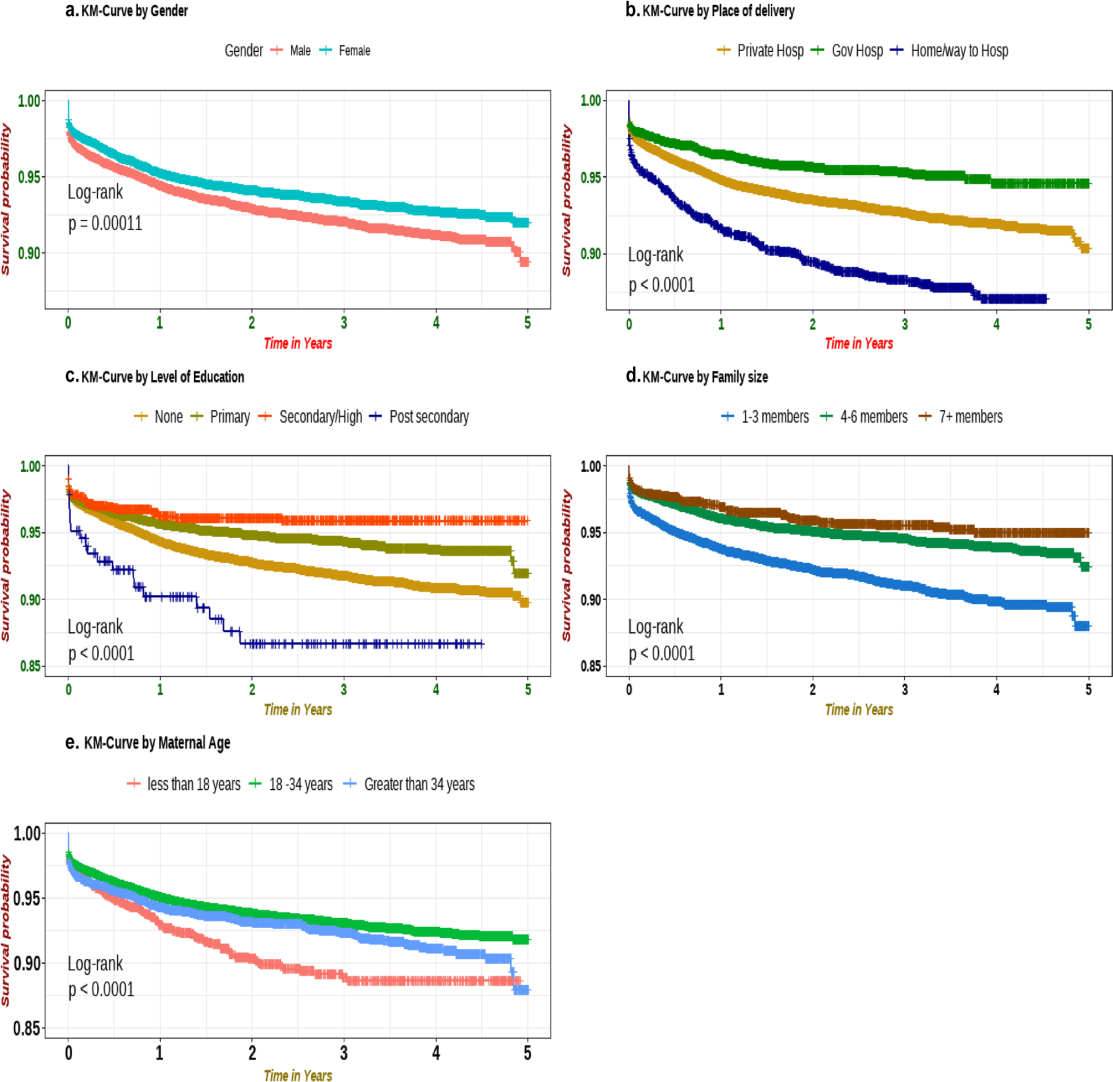
Kaplan Meier estimates showing child survival by selected epidemiological and health-related factors of participants in Siaya County, Kenya, using HDSS data from 2015 to 2020

We estimated the survival probability of children under the age of five years using the Kaplan-Meier curve estimate **(**Fig. 4a **– **e). The analysis of the Kaplan-Meier curves demonstrated that under-five children who are male, not delivered to hospitals, families with 1–3 members, advanced and young maternal age, and no maternal education have lower survival rates than the comparison group.

### Results of the multivariable analysis

Children who were female were 18% more likely to die compared to males (aHR 0.82; 95% CI 0.74–0.82) (Table 2). Children born to mothers with no education and primary education had a higher risk of death (aHR 1.29; 95% CI 1.14, 1.47) and (aHR 1.93; 95% CI 1.19,3.10), respectively. In addition, children born to mothers aged below < 18 years had a higher risk for mortality (aHR = 01.34; 95% CI 1.11, 1.62). Place of birth (aHR 1.5; 95% CI 1.29,1.77), ANC visits (aHR 2.1; 95% CI 1.14, 3.87), and type of latrine (aHR 1.22; 95% CI 1.04, 1.43) were also associated with childhood mortality.

**Table 2:** Cox proportional hazard model to identify factors associated with childhood mortality in Siaya County, Kenya using HDSS data 2015–2020

Risk factors	*Crude Hazard Ratio*		*Adjusted Hazard Ratio*	
	HR^1^	95% CI^1^	HR^1^	95% CI^1^
Maternal Level of Education				
*Primary*	1	—	1	—
*Secondary/High*	0.73	0.65, 0.82	0.77	0.68, 0.88
*Post Secondary*	0.55	0.41, 0.73	0.59	0.43, 0.82
* None*	1.73	1.14, 2.64	1.49	0.93, 2.37
Maternal Age				
*18–34 years*	1	—	1	—
*less than 18 years*	1.49	1.26, 1.77	1.34	1.11, 1.62
*Greater than 34 years*	1.16	1.01, 1.33	1.04	0.90, 1.21
Number of ANC visits				
*Above 4*	1	—	1	—
*None*	2.16	1.19, 3.93	2.1	1.14, 3.87
*One*	1.3	1.0, 1.70	1.11	0.84, 1.47
*Two*	1.4	1.18, 1.65	1.26	1.06, 1.49
*Three*	0.99	0.87, 1.12	0.94	0.83, 1.07
Place of birth				
*Government Hospital*	1	—	1	—
*Private hospital*	0.66	0.56, 0.78	0.7	0.60, 0.83
*Home/way to Hospital*	1.63	1.41, 1.87	1.51	1.29, 1.77
Method of cooking				
*Charcoal/Firewood*	1	—	1	—
*Gas/Paraffin/Stove*	0.65	0.42, 1.01	0.82	0.50, 1.32
Toilet type				
*Traditional pit*	1	—	1	—
*Modern latrine*	0.72	0.57, 0.89	0.84	0.67, 1.06
*Neighbor -No facility/Bush/field*	1.34	1.15, 1.55	1.22	1.04, 1.43
Child Gender				
*Male*	1	—	1	—
*Female*	0.82	0.74, 0.91	0.82	0.74, 0.91

*n* = 22,441; N events = 1,390; statistic.log = 158; p.value.log = 0.000; statistic.sc = 165; p.value.sc = 0.000; statistic.wald = 172; p.value.wald = 0.000; statistic.robust = 153; p.value.robust = 0.000; R² = 0.007; r.squared.max = 0.702; c-index = 0.592; c-index SE = 0.008; Log-likelihood = −13,494; AIC = 27,019; BIC = 27,098; No. Obs. = 22,441

^*1*^ *HR *Hazard Ratio, *CI *Confidence Interval

## Discussion

We observed that one in sixteen children died before the age of five in Western Kenya, a rate higher than the national average of one in twenty-four children. Maternal age, maternal education, number of ANC visits, toilet type, place of delivery, and gender were associated with childhood mortality. The overall cumulative survival probability of children was approximately above 90%, with the highest risk of death during the first month of life.

The high risk of death in the first month of life is consistent with findings from studies in Ethiopia and Nigeria [15, 20]. This may be attributed to factors such as low immunity and adverse fetal outcomes, including prematurity, birth asphyxia, and low birth weight, which play a crucial role in the susceptibility of neonates to preventable diseases [21]. Male infants were found to have a higher risk of dying before the age of 5 years compared to females, consistent with findings from other studies in LMICs [8, 22, 23]. This disparity could be due to the biological and genetic differences that make them more susceptible to many diseases that cause death [24]. Male children also exhibit lower disease resistance and greater vulnerability to complications of prematurity, contributing to a higher hazard risk of death [25].

Our findings revealed a significant association between maternal age and child survival, consistent with findings in previous studies [26]. Mothers with an age below 18 were at higher risk of child mortality, potentially due to their incomplete physical development, which can increase complications during pregnancy and childbirth [27]. Young mothers are also more likely to experience stigma, lower socioeconomic status, and lower educational attainment, all of which may negatively impact their health-seeking behavior and access to health services [28]. Furthermore, observed high outmigration among young mothers may have influenced the observed outcomes, as these mothers often relocate in search of better opportunities or due to social pressures, potentially disrupting continuity of care and access to critical maternal and child health services.

The observed lower under-five mortality rate among children born to mothers with higher education highlights the positive impact of maternal education on child survival outcomes, a finding consistent with previous studies [29, 30]. Studies suggest that educated mothers often have greater health literacy, enabling them to access health services earlier and make informed decisions about their child’s health [31, 32]. They are also more likely to be knowledgeable about various modern contraceptives, timely vaccination, proper hygiene, and proper nutrition, all of which contribute to improved child survival rates [12]. Educated mothers are often employed, providing them greater financial flexibility and independence to afford quality health care and seek treatment at well-equipped health facilities or with specialized doctors [33].

In this study, delivery in a health facility was significantly associated with lower child mortality, consistent with findings from other studies [34, 35]. Lower mortality among children born in health facilities may be attributed to comprehensive healthcare services provided to women and their children during labor and the postpartum period, as well as higher utilization of maternity services [36]. The WHO recommends a minimum of four visits during pregnancy, with the first visit in the first trimester [37]. In this study, children of mothers who attended fewer ANC visits faced a greater risk of death, aligning with results from a study in Ethiopia [16]. This positive impact of ANC services may be due to skilled healthcare workers educating mothers on pregnancy danger signs during routine checkups and administering routine vaccinations [38].

Household access to latrines was also associated with improved child survival, consistent with findings from other studies [39, 40]. This improvement in survival may be attributed to the health benefits of reduced exposure to human excreta, as shared or inadequately managed toilets can contribute to the spread of diseases and increased child mortality [41, 42]. In contrast, we found no significant association between the source of cooking fuel and under-five mortality, which diverges from most previous findings [43, 44]. The differences in the outcome may be attributed to the study setting, sample size, and geographical factors.

Our analysis has several strengths and limitations. The huge dataset provided valuable information for understanding under-five mortality and its predictors in Siaya County between 2015 and 2020. While the study did not utilize the national-level data, the study has highlighted a higher number of under-five deaths and is representative of population-level data. Limitations of this study include the potential biases or inaccuracies inherent in secondary data sources and Karemo HDSS’s use of a consecutive sampling approach, which excludes non-resident households, leading to selection bias. Additionally, some key variables were not included in the final analysis due to missing data, which may have affected the robustness of our findings and the generalizability of the results. The DIP in the final year of the survival curve coincides with the COVID-19 outbreak and could be attributed to multiple factors. The pandemic disrupted routine data collection, leading to underreporting or delays in recording cases. Additionally, health system disruptions, changes in healthcare-seeking behavior, and shifts in disease patterns due to COVID-19 mitigation measures might have influenced the numbers. Lastly, the extended follow-up period led to out-migration, which may have biased the estimates of mortality risk factors in this longitudinal study.

## Conclusion

Under-five mortality in Siaya County was markedly high at 63 deaths per 1,000 live births, significantly exceeding the national rate of 41.6 deaths per 1,000 live births. The risk of death was highest among the early neonates and declined with increasing age. Child death was more common among children of young mothers, mothers with no or low level of education, low Antenatal care (ANC) coverage, home deliveries, and households without access to latrines.

## Supplementary Information

Supplementary Material 1.

Supplementary Material 2.

## Data Availability

No datasets were generated or analysed during the current study.

## Abbreviations

ANC: Antenatal Care
CDC: Centre for Disease Control
COVID 19: Coronavirus disease 2019
HDSS: Health Demographic Surveillance System
HIV: Human Immunodeficiency virus
KEMRI: Kenya Medical Research Institute
LMCI: Lower and Middle Income countries

## References

[1] UN - IGME. Level & Trends in Child Mortality. 2018.

[2] Levels. Trends in child mortality: report 2022. New York: United Nations Children’s Fund (UNICEF); 2023.

[3] MOH Kenya. Kenya Community Health Strategy 2020–2025. 2020;1–44.

[4] Arora A, UNICEF DATA. 2024 [cited 2024 Oct 9]. Levels and trends in child mortality. Available from: https://data.unicef.org/resources/levels-and-trends-in-child-mortality-2024/

[5] Macharia RM. Factors Affecting Under Five Mortality In Coast And Central Kenya.

[6] Macharia PM, Giorgi E, Thuranira PN, Joseph NK, Sartorius B, Snow RW, et al. Sub National variation and inequalities in under-five mortality in Kenya since 1965. BMC Public Health. 2019;19(1):146. 30717714 10.1186/s12889-019-6474-1 PMC6360661

[7] Ettarh R, Kimani J. Determinants of under-five mortality in rural and urban Kenya. RRH [Internet]. 2012 Mar 13 [cited 2023 Aug 2]; Available from: https://www.rrh.org.au/journal/article/1812 22417123

[8] Fagbamigbe AF, Morakinyo OM, Balogun FM. Sex inequality in under-five deaths and associated factors in low and middle-income countries: a fairlie decomposition analysis. BMC Public Health. 2022;22(1):334. 35172780 10.1186/s12889-022-12679-y PMC8851802

[9] Nasejje JB, Mwambi H. Application of random survival forests in Understanding the determinants of under-five child mortality in Uganda in the presence of covariates that satisfy the proportional and non-proportional hazards assumption. BMC Res Notes. 2017;10(1):459. 28882171 10.1186/s13104-017-2775-6 PMC5590231

[10] Nasejje JB, Mwambi HG, Achia TNO. Understanding the determinants of under-five child mortality in Uganda including the Estimation of unobserved household and community effects using both frequentist and bayesian survival analysis approaches. BMC Public Health. 2015;15(1):1003. 26428635 10.1186/s12889-015-2332-y PMC4591593

[11] Wambugu MR. DETERMINANTS OF UNDER-5 MORTALITY IN KENYA DURING UPSURGE AND DECLINING TRENDS PERIOD.

[12] Patel N, Olickal JJ. Maternal and child factors of under-five mortality in India. Findings from NFHS-4. Clin Epidemiol Global Health. 2021;12:100866.

[13] Verhulst A, Prieto JR, Alam N, Eilerts-Spinelli H, Erchick DJ, Gerland P, et al. Divergent age patterns of under-5 mortality in South Asia and sub-Saharan africa: a modelling study. Lancet Global Health. 2022;10(11):e1566–74. 36088913 10.1016/S2214-109X(22)00337-0 PMC9588693

[14] Chapter 7. Longitudinal studies [Internet]. [cited 2024 Dec 9]. Available from: https://www.bmj.com/about-bmj/resources-readers/publications/epidemiology-uninitiated/7-longitudinal-studies

[15] Egbon OA, Bogoni MA, Babalola BT, Louzada F. Under age five children survival times in nigeria: a bayesian Spatial modeling approach. BMC Public Health. 2022;22(1):2207. 36443732 10.1186/s12889-022-14660-1 PMC9706907

[16] Oduse S, Zewotir T, North D. The impact of antenatal care on under-five mortality in ethiopia: a difference‐in‐differences analysis. BMC Pregnancy Childbirth. 2021;21(1):44. 33423662 10.1186/s12884-020-03531-5 PMC7798199

[17] Tesfa D, Tiruneh SA, Azanaw MM, Gebremariam AD, Engdaw MT, Kefale B, et al. Time to death and its determinants among under-five children in Sub-Saharan Africa using the recent (2010–2018) demographic and health survey data: country-based shared frailty analyses. BMC Pediatr. 2021;21(1):515. 34789187 10.1186/s12887-021-02950-3 PMC8597287

[18] Odhiambo FO, Laserson KF, Sewe M, Hamel MJ, Feikin DR, Adazu K, et al. Profile: the KEMRI/CDC health and demographic surveillance system-Western Kenya. Int J Epidemiol. 2012;41(4):977–87. 22933646 10.1093/ije/dys108 PMC12083774

[19] KNBS 2022. ECONOMIC SURVEY 2022. Kenya National Bureau of Statistics; pp. 425–30. Report No.: 978-9914-79987-3–3.

[20] Ayele DG, Zewotir TT, Mwambi H. Survival analysis of under-five mortality using Cox and frailty models in Ethiopia. J Health Popul Nutr. 2017;36(1):25. 28578680 10.1186/s41043-017-0103-3 PMC5455089

[21] Limaso AA, Dangisso MH, Hibstu DT. Neonatal survival and determinants of mortality in Aroresa district, Southern ethiopia: a prospective cohort study. BMC Pediatr. 2020;20(1):33. 31987037 10.1186/s12887-019-1907-7 PMC6983969

[22] Barford A, Dorling D, Davey Smith G, Shaw M. Life expectancy: women now on top everywhere. BMJ. 2006;332(7545):808. 10.1136/bmj.332.7545.808. PMID: 16601021; PMCID: PMC1432200. 16601021 10.1136/bmj.332.7545.808 PMC1432200

[23] Costa JC, Da Silva ICM, Victora CG. Gender bias in under-five mortality in low/middle-income countries. BMJ Glob Health. 2017;2(2):e000350. 29082002 10.1136/bmjgh-2017-000350 PMC5656133

[24] Chao F, Masquelier B, You D, Hug L, Liu Y, Sharrow D, et al. Sex differences in mortality among children, adolescents, and young people aged 0–24 years: a systematic assessment of national, regional, and global trends from 1990 to 2021. Lancet Global Health. 2023;11(10):e1519–30. 37734797 10.1016/S2214-109X(23)00376-5 PMC10522776

[25] Yaya S, Diarra S, Mabeu MC, Pongou R. The sex gap in neonatal mortality and the AIDS epidemic in sub-Saharan Africa. BMJ Glob Health. 2018;3(5):e000940. 30233834 10.1136/bmjgh-2018-000940 PMC6135478

[26] Ahinkorah BO. Under-5 mortality in sub-Saharan africa: is maternal age at first childbirth below 20 years a risk factor? BMJ Open. 2021;11(9):e049337. 34593494 10.1136/bmjopen-2021-049337 PMC8487196

[27] Cavazos-Rehg PA, Krauss MJ, Spitznagel EL, Bommarito K, Madden T, Olsen MA, et al. Maternal age and risk of labor and delivery complications. Matern Child Health J. 2015;19(6):1202–11. 25366100 10.1007/s10995-014-1624-7 PMC4418963

[28] Chirwa GC, Mazalale J, Likupe G, Nkhoma D, Chiwaula L, Chintsanya J. J Amo-Adjei editor 2019 An evolution of socioeconomic related inequality in teenage pregnancy and childbearing in Malawi. PLoS ONE 14 11 e0225374. 31747437 10.1371/journal.pone.0225374 PMC6867649

[29] Bado AR, Sathiya Susuman A. Women’s Education and Health Inequalities in Under-Five Mortality in Selected Sub-Saharan African Countries, 1990–2015. Carpenter DO, editor. PLoS ONE. 2016;11(7):e0159186. 10.1371/journal.pone.0159186 PMC4956109 27442118

[30] Wu H. The effect of maternal education on child mortality in Bangladesh. Popul Dev Rev. 2022;48(2):475–503.

[31] UNICEF. 2016. Maternal and Newborn Health Disparities. Unicef, Maternal and Newborn Health Disparities [Internet]. 8. Available from: 10.1016/S1386-9477(02)00298-9

[32] Andriano L, Monden CWS. The Causal Effect of Maternal Education on Child Mortality: Evidence From a Quasi-Experiment in Malawi and Uganda. Demography. 2019;56(5):1765–90. 10.1007/s13524-019-00812-3. PMID: 31591685; PMCID:PMC6797651. 31591685 10.1007/s13524-019-00812-3 PMC6797651

[33] Smith Greenaway E, Leon J, Baker DP. Understanding the association between maternal education and use of health services in ghana: exploring the role of health knowledge. J Biosoc Sci. 2012;44(6):733–47. 22377424 10.1017/S0021932012000041 PMC3590019

[34] Machio PM. Determinants of neonatal and under-five mortality in kenya: do antenatal and skilled delivery care services matter? Nairobi, Kenya: African Economic Research Consortium; 2017. p. 25. (AERC research paper).

[35] WHO. 2018. Definition of skilled health personnel providing care during childbirth: the 2018 joint statement by WHO, UNFPA, UNICEF, ICM, ICN, FIGO and IPA [Internet]. 2018. Available from: www.unfpa.org/sowmy

[36] Gage AD, Fink G, Ataguba JE, Kruk ME. Hospital delivery and neonatal mortality in 37 countries in sub-Saharan Africa and South Asia: An ecological study. Myers JE, editor. PLoS Med. 2021;18(12):e1003843. 10.1371/journal.pmed.1003843 PMC8635398 34851947

[37] WHO-RHR-18. 02-eng.pdf [Internet]. [cited 2024 Sep 30]. Available from: https://iris.who.int/bitstream/handle/10665/259947/WHO-RHR-18.02-eng.pdf

[38] Doku DT, Neupane S. Survival analysis of the association between antenatal care attendance and neonatal mortality in 57 low- and middle-income countries. Int J Epidemiol. 2017;46(5):1668–77. 29040531 10.1093/ije/dyx125 PMC5837573

[39] Adebowale SA, Morakinyo OM, Ana GR. Housing materials as predictors of under-five mortality in nigeria: evidence from 2013 demographic and health survey. BMC Pediatr. 2017;17(1):30. 28103828 10.1186/s12887-016-0742-3 PMC5248529

[40] Motsima T, Zuma K, Rapoo E. Levels and trends of Under-Five mortality in South Africa from 1998 to 2012. International Journal of Medical and Health Sciences. 2020;14(5). https://www.researchgate.net/publication/358348859_Levels_and_Trends_of_Under-Five_Mortality_In_South_Africa_from_1998_to_2012.

[41] Bitew BD, Woldu W, Gizaw Z. Childhood diarrheal morbidity and sanitation predictors in a nomadic community. Ital J Pediatr. 2017;43(1):91. 28985750 10.1186/s13052-017-0412-6 PMC5639577

[42] Makinde B, Francis A, Ayo S. Environmental Factors as Predictors of Childhood Mortality Experience in Nigeria. African Journal of Environmental Health Sciences. 5:23–34. https://www.researchgate.net/publication/331772218_Environmental_Factors_as_Predictors_of_Childhood_Mortality_Experience_in_Nigeria.

[43] Owili PO, Muga MA, Pan WC, Kuo HW. Cooking fuel and risk of under-five mortality in 23 Sub-Saharan African countries: a population-based study. Int J Environ Health Res. 2017;27(3):191–204. 28552005 10.1080/09603123.2017.1332347

[44] Naz S, Page A, Agho KE. Household air pollution from use of cooking fuel and under-five mortality: The role of breastfeeding status and kitchen location in Pakistan. Mortimer K, editor. PLoS ONE. 2017;12(3):e0173256. 10.1371/journal.pone.0173256 PMC5344381 28278260

