# Supplementary figures and images for: Survival probability and under-five mortality predictors in Western Kenya between 2015 and 2020
Source: BMC Public Health. 2025 Nov 10;25:3874. doi: 10.1186/s12889-025-25052-6 (PMC12604415; doi:10.1186/s12889-025-25052-6)

**Supplementary 1: Plot of variable completeness using Pareto Chart**


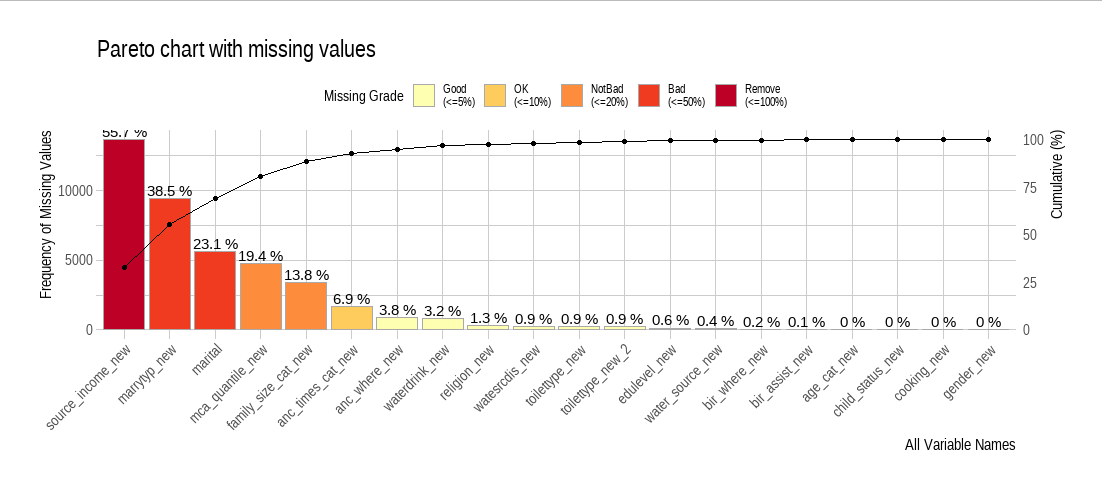

Supplement: Supplementary file 1 — Supplementary Material 1. [file 12889_2025_25052_MOESM1_ESM.docx]

**Supplementary 2: Proportion hazard assumption check**


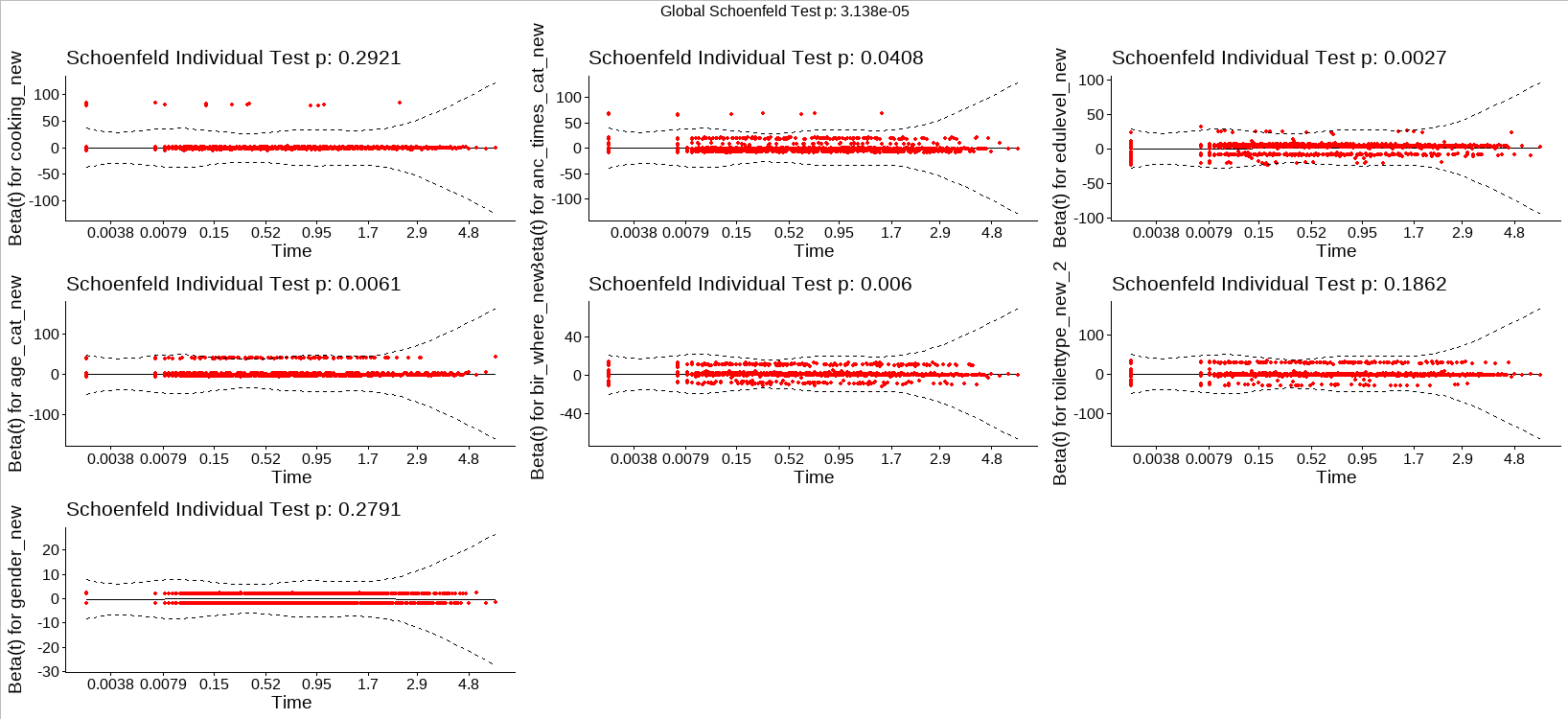

Supplement: Supplementary file 2 — Supplementary Material 2. [file 12889_2025_25052_MOESM2_ESM.docx]
